# Experimental observations and density functional simulations on the structural transition behavior of a two-dimensional transition-metal dichalcogenide

**DOI:** 10.1038/s41598-020-75240-0

**Published:** 2020-10-26

**Authors:** W. Liu, Z. Duan, C. Zhang, X. X. Hu, J. B. Cao, L. J . Liu, L. Lin

**Affiliations:** 1 School of Mechanical and Energy Engineering, Ningbo Tech University, Ningbo, 315100 China; 2 Ningbo Vorias Machinery Technology Co., Ltd, Ningbo, 315100 China; 3 Xingyu Electron (Ning Bo) Co., Ltd, Ningbo, 315514 China

**Keywords:** Electrical and electronic engineering, Mechanical engineering

## Abstract

In this work, we show an obvious evidence of nondestructive Raman spectra for the structural transition, i.e., the existence of a charge density wave (CDW) in monolayer 2H-TaS_2_, which can exhibit a much higher transition temperature than bulk and results in additional vibrational modes, indicating strong interactions with light. Furthermore, we reveal that the degenerate breath and wiggle modes of 2H-TaS_2_ originated from the periodic lattice distortion can be probed using the optical methods. Since recently several light-tunable devices have been proposed based on the CDW phase transition of 1 T-TaS_2_, our study and in particular, the theoretical results will be very helpful for understanding and designing electronic devices based on the CDW of 2H-TaS_2_.

## Introduction

Charge density waves (CDW), structural transitions from a normal to a distorted phase, have been extensively studied for many decades^1^. They are characterized by the competing influences of the energy cost associated with distortion of the crystal structure and liberation due to the opening of an electronic energy gap. CDW transitions are known to occur in wide variety of materials, a prominent example being layered transition-metal dichalcogenides (TMDs), which have received much attention due to the wealth of their novel optical and electronic properties. The properties of TMDs often show a strong dimensional dependence, including the CDW phase transition^2,3^. It has been suggested that it is possible to tune the CDW transition temperature of thin TMDs through dimensionality, electrostatic gating or strain engineering^4^. This would enable the quantum phase transitions to be controlled in a manner that is compatible with current semiconductor technology. Tantalum disulfide, TaS_2_, is an archetypal TMD material which exhibits CDWs. It consists of layers stacked by weak van der Waals bonding, with each covalently bound layer typically consists of a sheet of hexagonally arranged Ta atoms sandwiched between two S layers. 1 T-TaS_2_ shows a $\sqrt {13} \times \sqrt {13}$ CDWs below 540 K. Upon cooling from 540 K, 1 T-TaS_2_ undergoes several CDW transitions. It changes from incommensurate to nearly commensurate at 350 K and then to commensurate at 180 K^5^. Bulk 2H-TaS_2_ forms a 3 × 3 commensurate CDW at low temperature^6^. It undergoes an incommensurate in-plane CDW transition at about 78 K^7,8^ and a superconducting transition at about 0.8 K^9^. Previous studies of 1 T-TaS_2_ have shown the importance of dimensionality for the CDW phase transitions^3,8^. It was found that as the thickness was reduced, the transition from the nearly commensurate to the commensurate CDW phase shifts toward lower temperatures during cool-down and suddenly vanished for a critical thickness. However, a recent study showed that the commensurate CDW phase is the ground state even for monolayer 1 T-TaS_2_^10^. CDW analysis of monolayer of 1 T and 1H of TaS_2_ has been reported, and the complexity of the CDWs suggests that unexplored modes may be excited for different external driving frequencies^11^. While the thickness-dependent CDW in 1 T-TaS_2_ has been intensively studied, less is known about the CDW structure in thin exfoliated 2H-TaS_2_, especially the CDW structure in mono- and few-layer 2H-TaS_2_. It is interesting to note that notably mono- and few-layer 2H-TaS_2_ exhibits strong interactions with light^12^. Moreover, CDWs in TaS_2_ spontaneously breaking lattice symmetry through periodic lattice distortion, and electron–electron and electron–phonon interactions may lead to a new type of electronic structure. Thus, optical methods provide a non-destructive and easy way to probe the electronic structure and transitions in 2H-TaS_2_.

Here, in combination with density functional simulations, we demonstrated that CDWs can exist in exfoliated monolayer 2H-TaS_2_ and the transition temperature is much higher than that of the bulk. A new peak appears at 155 cm^−1^ below transition temperature, which corresponds to the breath mode and wiggle mode of the CDW of 2H-TaS_2,_ suggesting that the charge density wave transition and periodic lattice distortion can be probed and determined by optical methods, such as temperature dependent Raman scattering.

## Methods

### Sample preparation and characterizations

TaS_2_ nanosheets with different thicknesses were mechanically exfoliated from bulk 2H-TaS_2_ purchased from HQ Graphene. Optical color contrast and Seiko SPI3800N Atomic Force Microscopy (AFM) measurements were combined to identify the thicknesses of the nanosheets. The temperature dependent Raman spectra were taken using a Bruker Senterra confocal spectrometer with an excitation wavelength of 532 nm. High-resolution transmission electron microscopy (HRTEM) was performed using a JEOL-TEM.

### Phonon dispersion calculation

The phonon dispersion calculations for bulk and monolayer of 2H-TaS_2_ were carried out using a supercell approach^13^ with the PHONOPY code^14^. Before executing Phonopy package^15^, the fully relaxed structures were obtained from the VASP relaxation procedure. To optimize the bulk and monolayer 2H-TaS_2_ by employing VASP, the lattice parameter for the bulk unit cell was set as 2.85 Å, according to the HRTEM results, the energy cut of plane wave expansion was set to 500 eV, the k-points adopted from Monkhorst–Pack method were set to be 16 × 16 × 4 for bulk structure and 16 × 16 × 1 for monolayer structure, and the energy and atomic force convergence criteria for self-consistent was set to be 10^−9^ eV and 10^−6^ eV/Å, respectively. Moreover, the Van der Waal force interaction has been taken into account. For phonon dispersion calculations, the size of supercell has been chosen to be 4 × 4 × 4 for bulk and 4 × 4 × 1 for monolayer 2H-TaS_2_, respectively. In addition, we chose a 2 × 2 × 1 supercell extension for the calculating phonon dispersion of a 3 × 3 × 1 unit cell structure where the CDW phase of the monolayer 2H-TaS_2_ existed. Here it should be pointed out that the smearing parameter *σ* = 0.03 eV was used for the calculations of spectra since the depth of the negative peaks in the preceding spectra were smearing dependent.

## Results and discussion

**Figure 1:**
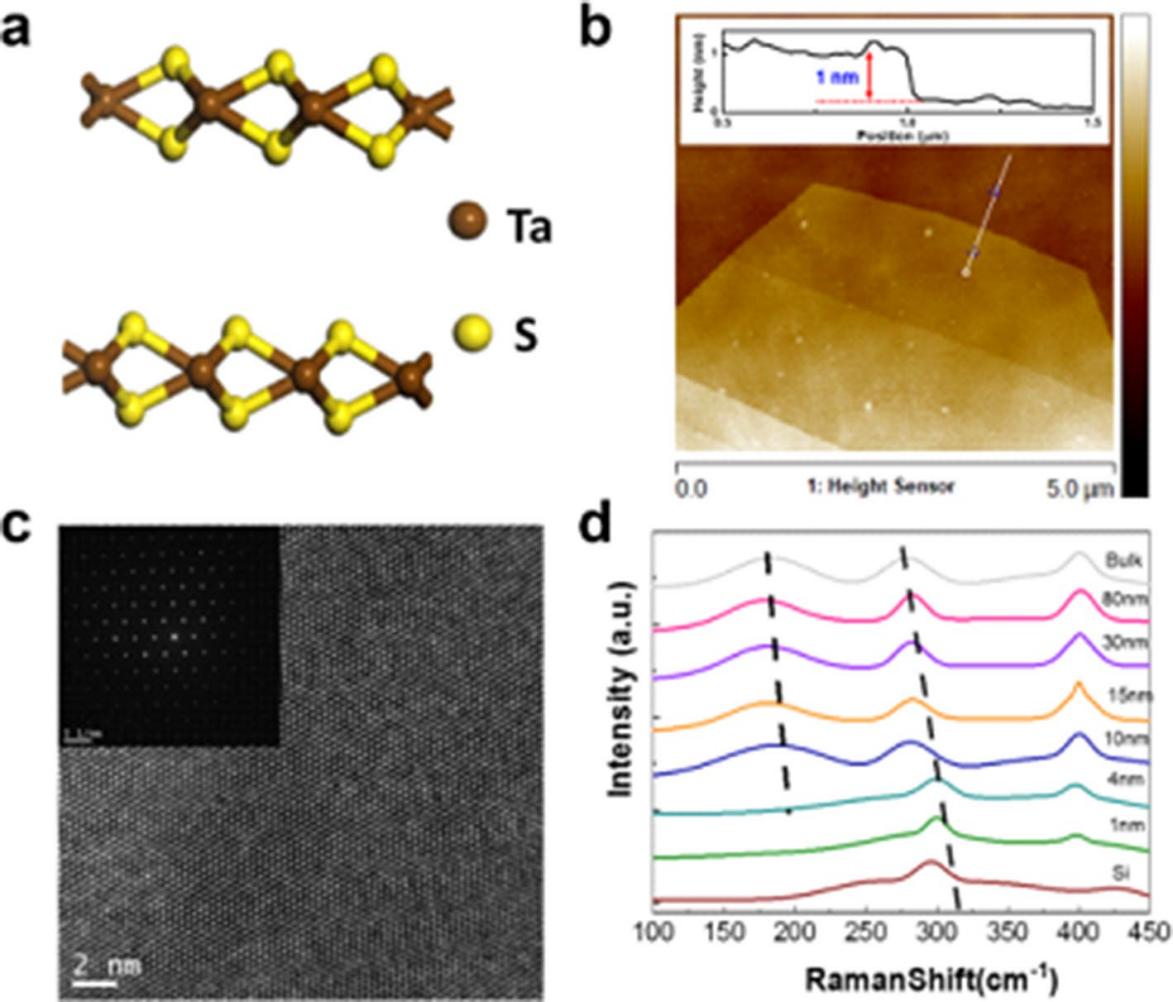
TaS_2_ characterizations. (**a**) Schematic drawing of the atomic structure of TaS_2_. (**b**) AFM image of mechanically exfoliated TaS_2_ flakes with a thickness about 1 nm. (**c**) HRTEM of TaS_2_ flakes. Inset: SAED of TaS_2_. (**d**) Raman spectra of mechanically exfoliated TaS_2_ flakes with various thicknesses. The excitation wavelength is 532 nm.

In the study, 2H-TaS_2_ nanosheets of different thicknesses (from 1 to over 100 nm) were exfoliated from a commercially grown 2H-TaS_2_ single crystal and then transferred onto SiO_2_/Si substrates by Scotch tape. Figure 1a shows the atomic structure of 2H-TaS_2_, where Ta atoms are in trigonal prismatic coordination with the S atoms. Atomic force microscope (AFM), transmission electron microscopy (TEM) and Raman spectroscopy were used to investigate the thickness and quality of the exfoliated 2H-TaS_2_ nanosheets. As shown in Fig. 1b, the smooth AFM image of the exfoliated 2H-TaS_2_ nanosheet indicates the layered structure. The cross-sectional height reveals that the thickness of the exfoliated TaS_2_ film is about ~ 1 nm. Here it should be indicated that all the thicknesses are only determined to the nearest nanometer (e.g., the “1 nm” sample is really 1.2 nm). The high resolution TEM image (Fig. 1c) and corresponding selected area electron diffraction (inset of Fig. 1c) of TaS_2_ demonstrate the single crystal hexagonal lattice structure and high quality of the exfoliated sample. It is also noted that the lattice parameter 2.85 Å indicates that the exposed surface is the (100) plane of 2H-TaS_2_. Figure 1d displays the Raman spectra of 2H-TaS_2_ for various thicknesses, excited by 532 nm laser line in ambient environment. The Raman spectra of thick 2H-TaS_2_ is consistent with previous reports^16,17^, and the Raman data of the ultrathin sample is shown here for the first time to the best of our knowledge. A_1g_ (~ 400 cm^−1^ for bulk TaS_2_) and $E_{2g}^{1}$ (~ 280 cm^−1^ for bulk TaS_2_) modes are observed in both ultrathin and bulk TaS_2_. The other two modes ($E_{1g} ,E_{2g}^{2}$) could not be detected either because of selection rules for our scattering geometry ($E_{1g}$) or because of the limited rejection of the Rayleigh scattered radiation ($E_{2g}^{2}$). Remarkably, a strong band peaking at ~ 180 cm^−1^ is observed for thick samples due to second-order scattering. With increasing layer number, the interlayer Van der Waals force in 2H-TaS_2_ suppresses the out-of-plane vibration, so both the second-order scattering and $E_{2g}^{1}$ mode are stiffened (blue shift). While the red shift of the A_1g_ mode indicates that long-range Columbic interlayer interactions may dominate variation of Raman mode, which is consistent with many other 2D materials^18^. Noticeably, the Raman data of the thin sample (< 4 nm) shows two significant differences with respect to the thicker samples, where the second-order scattering peak degenerates and the $E_{2g}^{1}$ mode shows a dramatic red shift.

**Figure 2:**
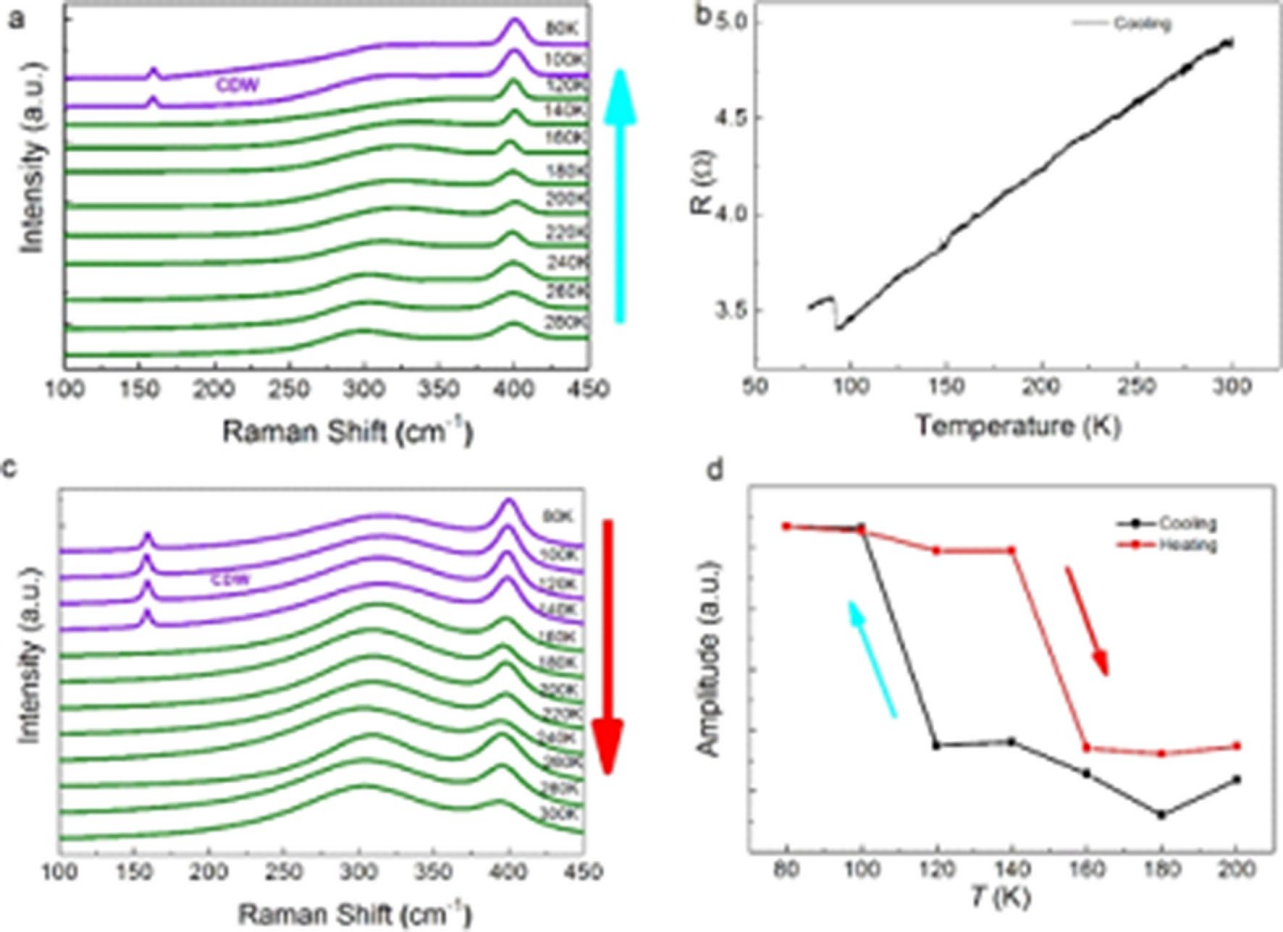
Probing CDW in monolayer TaS_2_. (**a**) Raman spectra for monolayer TaS_2_ acquired during the cooling cycle. (**b**) Resistivity measurement shows a temperature-induced phase transition. (**c**) Raman spectra for the same monolayer TaS_2_ acquired during heating cycle. (**d**) Temperature dependence of the Raman intensity for Raman mode at 400 cm^−1^.

Figure 2a presents the temperature evolution of the Raman spectra of a 2H-TaS_2_ monolayer measured at the same position during a cooling cycle. With decreasing temperature, the ${\varvec{E}}_{{{\mathbf{2}}{\varvec{g}}}}^{{\mathbf{1}}}$ mode shows a red shift. Interestingly, apart from the peaks of the A_1g_ and the ${\varvec{E}}_{{{\mathbf{2}}{\varvec{g}}}}^{{\mathbf{1}}}$ modes, a new peak appears at ~ 155 cm^−1^, when the temperature is below 100 K. Bulk 2H-TaS_2_ undergoes a phase transition at 75 K and the distorted CDW phase is formed below the transition temperature, *T*_*c*_^19^. The appearance of the new peak may be due to the formation of the CDW in monolayer 2H-TaS_2_ at low temperature. Here it is noted that the 280 K spectrum shown in Fig. 2a is not identical to the 1 nm spectrum shown in Fig. 1d, e,g, the relative heights of the two peaks differ in the two figures, implying a dependence of temperature cycling. To confirm this, Fig. 2b plots the temperature-dependent electrical resistance curve for monolayer 2H-TaS_2_ during cooling. A sudden jump in resistance is observed at 93 K, indicating the CDW phase transition occurs even in monolayer 2H-TaS_2_ and the transition temperature is higher than that for the bulk. The increased *T*_*c*_ may be due to the reduced dimensionality, which enhances electron–phonon coupling and has been observed in other two-dimensional CDWs^20,21^. Figure 2c further shows the Raman spectra of the same monolayer 2H-TaS_2_ during the heating cycle. Remarkably, the new peak can be clearly observed even at 140 K. The observed thermal hysteresis effect may be due to the presence of hidden CDW states^8,22^ or the roughness of the substrate^23^. Figure 2d summarizes the intensity of the A_1g_ mode as a function of temperature. It is found that the intensity of the A_1g_ mode also shows a similar thermal hysteresis effect. We also measured 2H-TaS_2_ flakes of other thicknesses (figure S1). Similar effects were observed. We would like to stress that our work is the first to demonstrate that CDWs can exist in monolayer 2H-TaS_2_.

**Figure 3:**
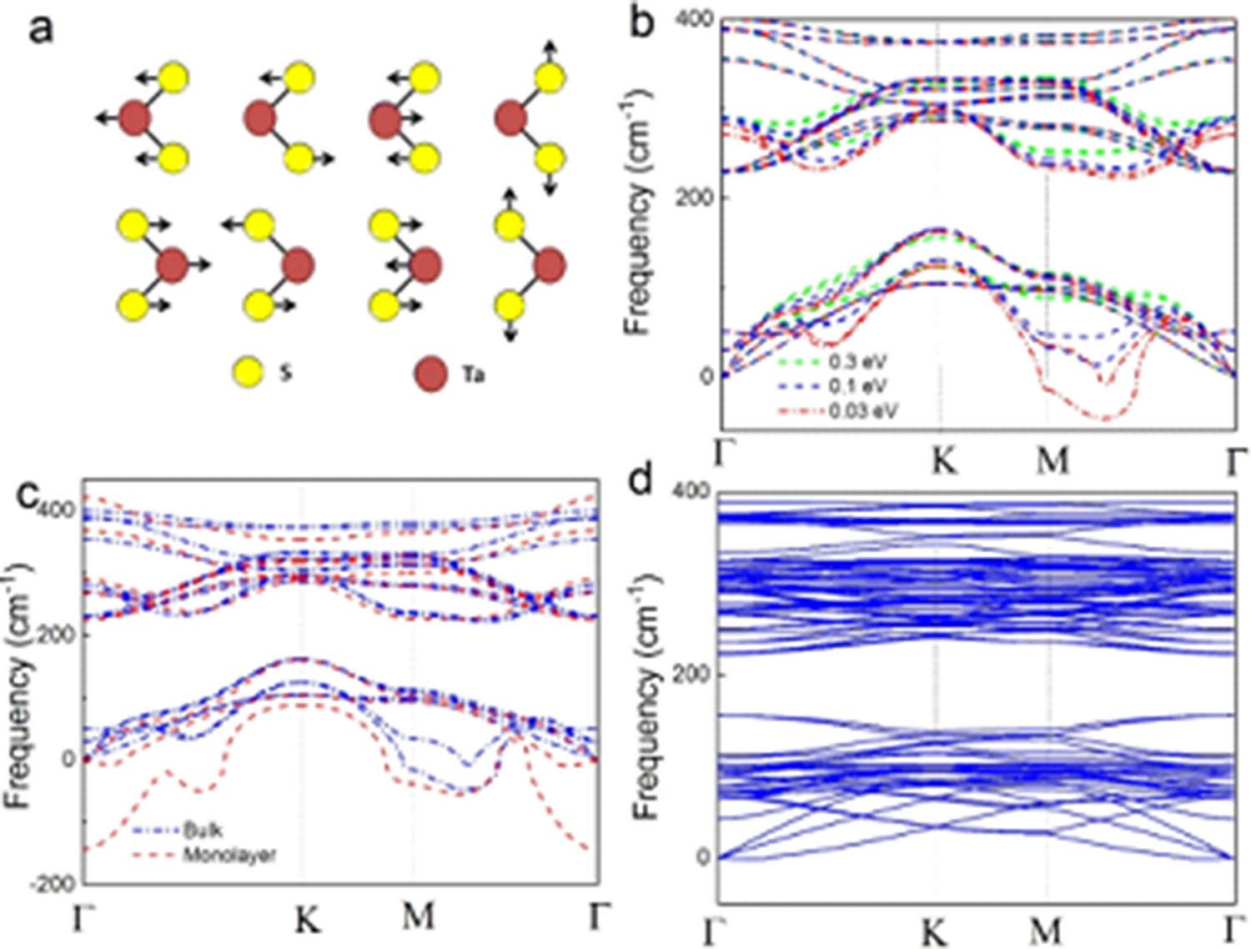
Phonon dispersion of TaS_2_. (**a**) Schematic of E^1^_2g_, E_1g_, E^2^_2g_, and A_1g_ Raman active modes of bulk 2H-TaS_2_. (**b**) Phonon dispersion of bulk 2H**-**TaS_2_ as a function of electronic smearing parameter σ. (**c**) Phonon dispersion of bulk and monolayer 2H**-**TaS_2_ with a smearing parameter σ = 0.03 eV. (**d**) Phonon dispersion of monolayer 2H**-**TaS_2_ with a 3 × 3 × 1 unit cell.

To further confirm that the additional peaks observed at low temperature originate from the formation of a CDW, we calculated the phonon dispersion for the lattice dynamics of bulk and monolayer 2H-TaS_2_ with and without the CDW phase based on density functional theory^24–26^ and the PHONOPY code^13–15^. Details of the simulation can be found in “Methods” section. The bulk has a crystal symmetry belonging to the D_6h_ space group. Two molecular units of TaS_2_ compose a unit cell of bulk 2H-TaS_2_, resulting in a total of 18 phonon bands. From the symmetry point of view, the irreducible representations of vibrational modes for bulk 2H-TaS_2_ of the D_6h_ space group are: A_1g_ + 2B_2g_ + E_1g_ + 2E_2g_ + 2A_2u_ + B_1u_ + 2E_1u_ + E_2u_, where E^1^_2g_, E_1g_, E^2^_2g_, and A_1g_ are Raman active modes^18^, as shown in Fig. 3a. Figure 3b shows the phonon dispersion of bulk 2H-TaS_2_. Among them, three bands belong to acoustic branches and fifteen branches belong to optical branches, additionally, an obvious indirect phonon frequency gap of about 60 cm^−1^ exists between the acoustic and optical branches. Significantly, with a smearing parameter σ = 0.03 eV, there is a segment of acoustic branches which has negative phonon frequencies, approximately less than 50 cm^−1^ along the MΓ direction in bulk 2H-TaS_2_. A similar phenomenon, with negative phonon frequencies, also occurs in monolayer 2H-TaS_2_, as shown in Fig. 3c, where the maximum negative frequency approaches to 150 cm^−1^ along the M-Γ-K direction. The calculated results show that the bulk and monolayer 2H-TaS_2_ are mechanically unstable in their ground states. In VASP calculations, the smearing parameters were used to accelerate the convergence in electronic self-consistent calculations, and from physical point of view, the abrupt change in Fermi–Dirac distribution can be smeared out by changing the magnitude of σ in the ground state. As a result, this smearing parameter physically represents the electronic temperature and can qualitatively affect the phonon properties of the materials by considering temperature effects. Manifestly, from Fig. 3b,c, the dependence of the phonon bands of bulk and monolayer of 2H-TaS_2_ on the smearing parameter demonstrates that the large negative phonon frequencies along the acoustic branches will eventually be overshadowed, and become wholly positive frequencies as smearing increases with temperature.

The emergence of negative phonon frequencies along the M-Γ-K direction in both bulk and monolayer 2H-TaS_2_ provide a clue to obtain the phonon dispersion of monolayer 2H-TaS_2_ in the CDW phase. Physically, negative phonon frequencies represent an unstable mechanical material structure, so in order to get rid of these negative phonon frequencies or to obtain the stable structure of the monolayer 2H-TaS_2_, the unit cell should be extended along the ΓM direction. Significantly, such an extension of the unit cell along the ΓM direction basically behaves like the experimentally observed CDW phase in bulk 2H-TaS_2_, where the CDW phase is close to a supercell of 3 × 3 × 1 of the unit cell structure^27,28^. Figure 3d shows the phonon dispersion plot for monolayer of 2H-TaS_2_ with a 3 × 3 × 1 unit cell. The negative phonon frequencies have completely vanished along the path passing through high symmetry points. This firmly demonstrates that a 3 × 3 × 1 unit cell structure is mechanically stable. Secondly, two distinct phonon frequencies emerge at about 150 cm^−1^ at the Γ point that are not observed for bulk 2H-TaS_2_. From the above simulation results, one can confirm that the CDW phase of monolayer 2H-TaS_2_ truly and stably exists in a 3 × 3 × 1 unit cell structure. Moreover, the two CDW-induced frequencies at ~ 155 cm^−1^ from our numerical simulation coincide very well with the experimental results of the Raman spectra, as shown in Fig. 2.

To summarize this argument, for 2H-TaS_2_ with a 1 × 1 × 1 unit cell, one cannot get CDW peaks at 155.6 cm^−1^ as observed in experiments. However, for 2H-TaS_2_ with a 3 × 3 × 1 unit cell, two CDW modes appears at frequency 155.6691 cm^−1^ and 155.6718 cm^−1^ from ab initio calculations, which are very close to experimental observations near 155 cm^−1^. From the animations, there are two different modes near 155 cm^−1^, the Ta atoms will come closer and farther away from the center sulfur atoms like a breathing action (Breath mode), while for the other CDW mode the Ta atoms wiggle back and forth around the central S atoms like a wiggling motion (Wiggle Mode)^29^. The breath CDW mode and wiggle CDW mode can be viewed as degenerate modes.

**Figure 4:**
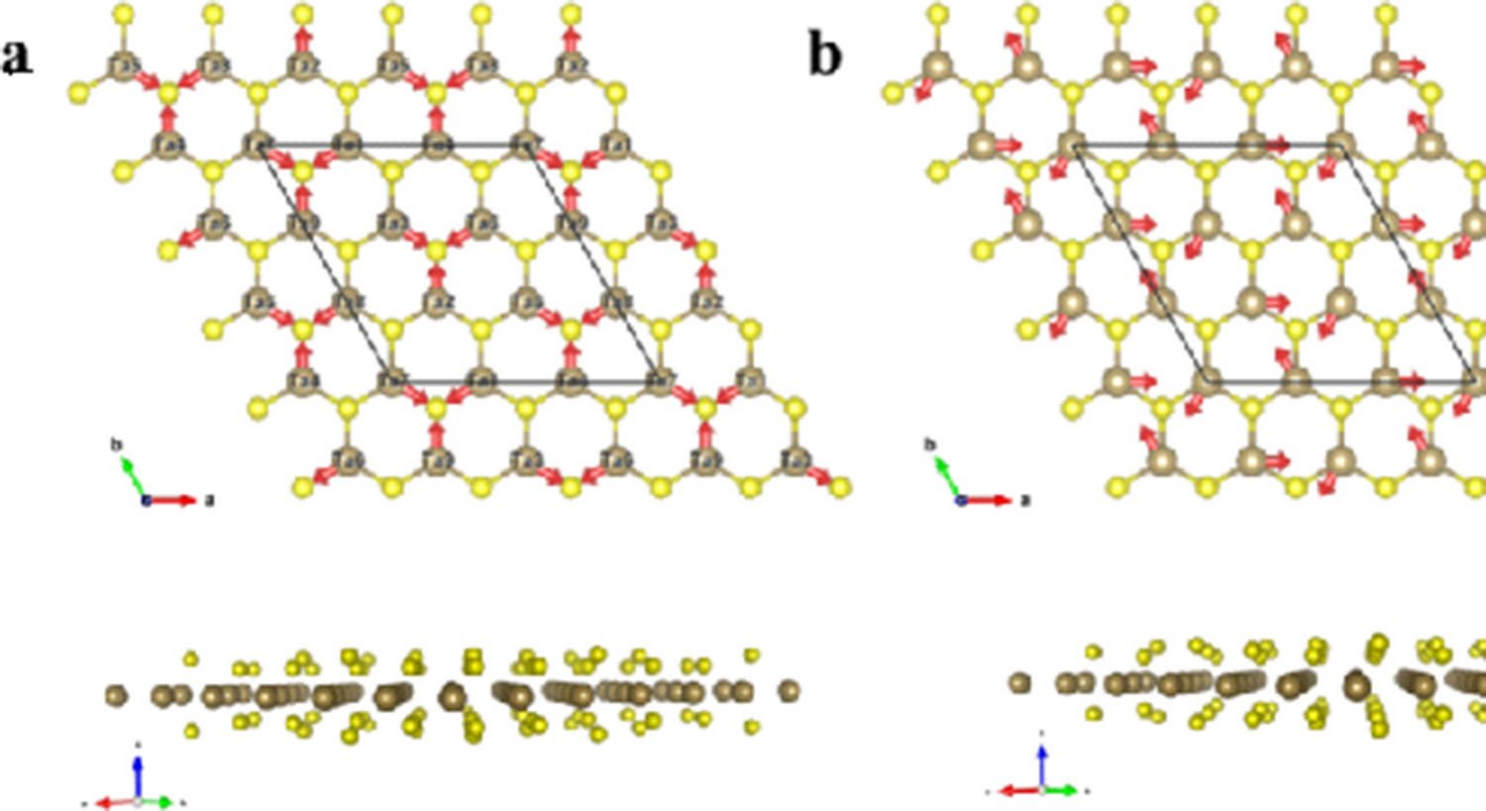
Degenerate modes of CDW TaS_2_. Top- and side-views of the atom displacements for the degenerate (**a**) breath CDW mode and (**b**) wiggle CDW mode. The yellow color denotes the sulfur atoms and the brown color denotes the tantalum atoms.

**Figure 5:**
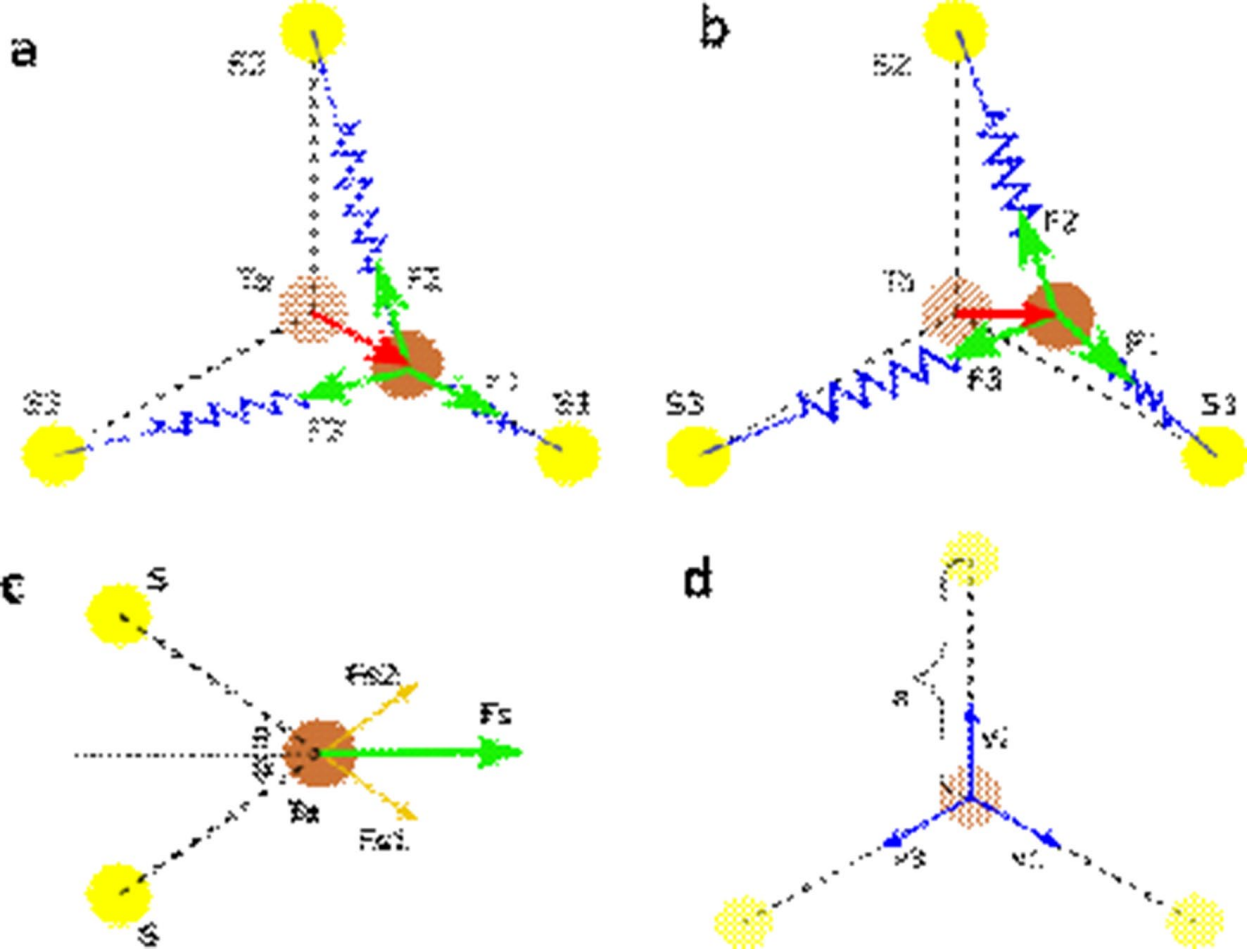
Origin of degenerate modes of CDW TaS_2_. The top view of the force contributions from the nearby atoms for (**a**) breath mode and (**b**) wiggle mode. (**c**) The side view of the force contributions from two nearby sulfur atoms on top of each other. (**d**) The definition of in-plane vectors along the un-distorted lattice bonds.

It is worth discussing the origin of the degeneracy of the oscillation frequency for these two modes. To clearly show the vibrational patterns it is necessary to consider a cell size 3 times larger in both the *a* and *b* directions for the CDW 3 × 3 × 1 unit cell. Figure 4 offers a top-down view of the lattice with the direction of the atomic displacements shown for both the breath and wiggle modes. The arrows denote the directions along which the atoms oscillate back and forth. Realistically, both the tantalum (Ta) and sulfur (S) atoms oscillate, however the displacement is comparatively small for S, thus we need only consider the movement of the Ta. Although the Ta atoms apparently oscillate in very different manners for the breath and wiggle modes, they are indeed the same at the larger scale if consider the collective motion of individual nearest atom triangular sub-units defined by 3 S atoms around a Ta atom. This equivalence can be more clearly recognized if we shift the origin of the wiggle mode by $- \frac{1}{3}b$. Therefore, to analyze the origin of the degenerate frequencies, we only need to focus on the triangular cells, as shown in Fig. 5. The oscillations are regulated by the force exerted by the restorative potentials and can effectively be treated as a system or lattice of Ta atoms connected by several springs. As the lowest order approximation, we need consider only the nearest S atoms effecting spring forces from the Ta-S bonds, and all the spring constants are the same since the S atoms are equidistant. There are 6 S atoms around each Ta atom. Since the 2H-TaS_2_ structure has a honeycomb structure, from the top view 3 S atoms situated are above the other 3 S atoms, thus the movements of Ta atoms for both modes are in-plane, and the S atoms can be further simplified by treating them as 3 in-plane atoms. If the spring constant between the actual S atoms and the Ta atom is *K*, the combined force of by the two vertically aligned S atoms is $\left| {{\mathbf{F}}{\text{s}}} \right| = \left| {{\mathbf{F}}{\text{s}}1 + {\mathbf{F}}{\text{s}}2} \right| = 2\cos \theta K\Delta x$ (Fig. 5c), where $\left| {{\mathbf{F}}{\text{s}}1} \right| = \left| {{\mathbf{F}}{\text{s}}2} \right| = K\Delta x$ and Δ*x* is the atomic displacement of Ta from its equilibrium point. The contribution from two vertically aligned S atoms is equivalent to an in-plane atom with effective spring constant k = 2cos*θK*.

For the breath mode shown in Fig. 5a, the Ta atoms oscillate along the line connecting a Ta and S atom. The net force exerted on the displaced Ta atom is the sum of the spring force from the 3 neighboring atoms, which is **F** = **F**1 + **F**2 + **F**3, where 1, 2, and 3 label the three simplified S atoms and their respective directions. The Ta atom is shifted toward S1 by Δx, and thus **F**1 =  − kΔx**v1**, where **v1** is the unit vector toward S1 (Fig. 2d). Considering the lowest order approximation, the spring constant is assumed to be isotropic. Therefore, the effective displacement with respect to S2 is $\left| {{\text{R}}_{{{\text{Ta}} - {\text{S}}2}} } \right| - a = \sqrt {a^{2} + \Delta x^{2} - 2a\Delta x\cos \Phi } - a\,\sim \,\Delta x/2$, and its direction is along **v2** since the displacement Δ*x* is very small. Similarly, the effective displacement from S3 atom is Δ*x*/2 along **v3**. Therefore, their spring forces are ${\mathbf{F}}2 = \frac{{{\text{k}}\Delta {\text{x}}}}{2}{\mathbf{v}}2$ and ${\mathbf{F}}3 = \frac{{{\text{k}}\Delta {\text{x}}}}{2}{\mathbf{v}}3$, respectively. The total spring force on the Ta atom is therefore ${\mathbf{F}} = - \frac{{3{\text{k}}}}{2}\Delta {\text{x}}{\mathbf{v1}}$. Assuming the Ta atom has mass m, the oscillation frequency for breath mode is obtained to be ${\upomega = }\sqrt {\frac{3k}{{2m}}}$. Displacement along **v2** and **v3** would be equivalent. For the wiggle mode Ta atom displacement is not directed towards the S atoms but rather towards the next adjacent Ta atom. Performing the same geometric analysis in this case, the displacement is along the x direction, the force due to the 3 three simplified S atoms are ${\mathbf{F}}1 = \frac{{\sqrt 3 {\text{k}}}}{2}\Delta {\text{x}}{\mathbf{v1}}$, **F**2 = 0, and ${\mathbf{F}}3 = \frac{{\sqrt 3 {\text{k}}}}{2}\Delta {\text{x}}{\mathbf{v3}}$. Therefore, the resultant force ${\mathbf{F}} = ( - \frac{{3{\text{k}}}}{2}\Delta {\text{x}}){\mathbf{x}}$, where **x** is unit vector along the lattice vector **a1**. Therefore, the oscillation frequency for the wiggle mode is still ${\upomega = }\sqrt {\frac{3k}{{2m}}}$. Thus to concluded, at the lowest order both the breath and wiggle modes are degenerate, displaying the same oscillation frequency. However, here we have assumed that the three Ta-S bonds have same bond energies, thus conferring the degeneracy of the breath and wiggle modes. However, internal effects (e.g. defects) or external effects (e.g. applied stress) can introduce anisotropy into the CDW modes, the degeneracy can be lifted, and three Ta atoms will vibrate incoherently and several nearby peaks should be observed around 155.6 cm^−1^.

## Conclusions

Charge density waves can exist in exfoliated monolayer 2H-TaS_2_ and the transition temperature can reach 140 K, which is much higher than that of the bulk. Moreover, the degenerate breath and wiggle modes of 2H-TaS_2_ originated from the periodic lattice distortion have been probed by the optical methods. Our results open an avenue to investigate charge density wave phase in two-dimensional transition-metal dichalcogenides and will be helpful for understanding and designing devices based on charge density waves.

## Supplementary information

Supplementary Figure.

## Data Availability

All data, models, and code generated or used during the study appear in the submitted article.
