# Supplementary material for: Experimental observations and density functional simulations on the structural transition behavior of a two-dimensional transition-metal dichalcogenide
Source: Sci Rep. 2020 Oct 26;10:18255. doi: 10.1038/s41598-020-75240-0 (PMC7588463; doi:10.1038/s41598-020-75240-0)
Supplement: Supplementary file 1 — Supplementary Figure. [file 41598_2020_75240_MOESM1_ESM.doc]

**Supplementary Figure**

**
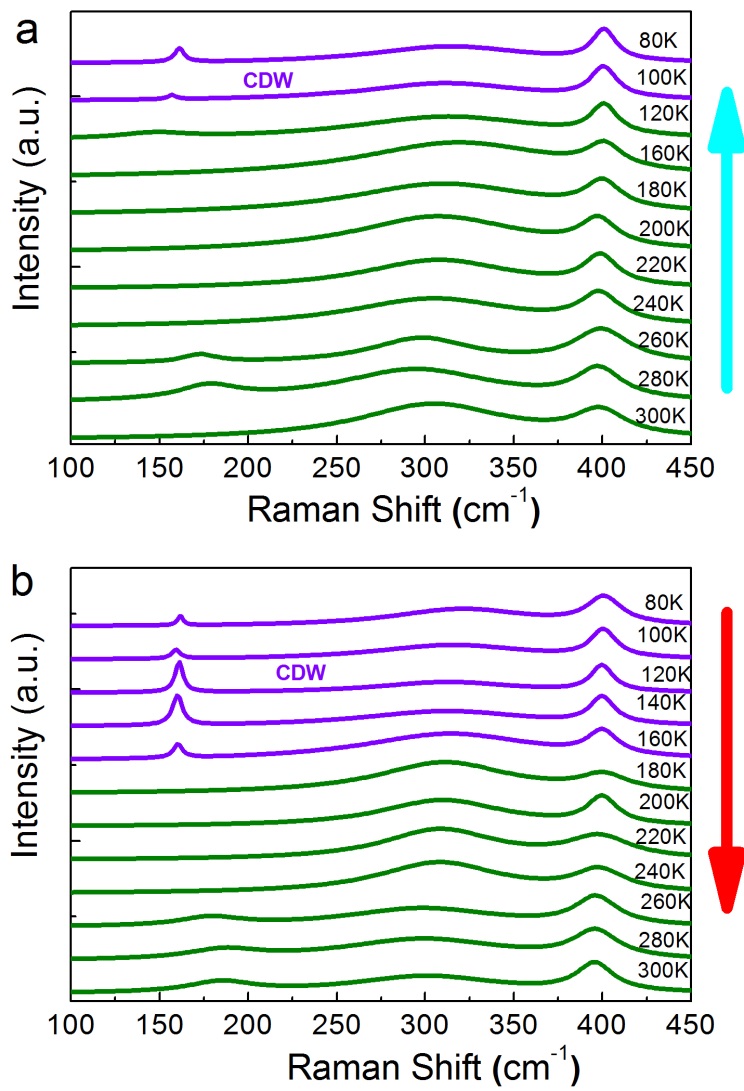
**

**Figure S1.** Probing CDW in 10 nm thick TaS_2_. Raman spectra for 10 nm thick TaS_2_ acquired during (a) cooling and (b) warming cycles.
